# The Relationship Between Contextual and Dispositional Variables, Well-Being and Hopelessness in School Context

**DOI:** 10.3389/fpsyg.2020.533815

**Published:** 2020-09-11

**Authors:** Caterina Buzzai, Luana Sorrenti, Susanna Orecchio, Davide Marino, Pina Filippello

**Affiliations:** Department of Clinical and Experimental Medicine, University of Messina, Sicily, Italy

**Keywords:** well-being, academic achievement, school hopelessness, dispositional variables, contextual variables, adolescent students

## Abstract

The literature’s interest has been focused on the study of well-being or depression. However, there has been little research that investigates the relationship between well-being and hopelessness (HPL) and the underlying contextual and dispositional variables. Therefore, the aim of the present study was to examine the relationship between some contextual (need-supportive interpersonal behavior and need-thwarting interpersonal behavior) and dispositional variables (dispositional optimism, positive/negative affectivity, explanatory style), academic achievement, general well-being, and school HPL in adolescent students. The results showed that general well-being was positively predicted by need-supportive interpersonal behavior, dispositional optimism, positive affectivity, and adaptive explanatory style (attribution to commitment in the school context), while it was negatively predicted by negative affectivity. Meanwhile, school HPL was positively predicted by need-thwarting interpersonal behavior, negative affectivity, dysfunctional explanatory style (attribution to luck in the school context), while it was negatively predicted by attribution to commitment in the school context and academic achievement. These results provide useful data for the implementation of well-being promotion and school HPL prevention. The implications are discussed as follows.

## Introduction

Integrating different theoretical approaches on wellness, Seligman (2011) developed “Positive Psychology 2.0.” The new theory (Seligman, 2011) considers well-being a complete realization of individuals’ potential and optimal functioning (flourishing) and is based on the interdependence of five factors (PERMA) that constitute and define it: (a) Positive Emotion (P), which is the possibility of experimenting and focusing on positive emotions without dwelling too much on negative events and facing one’s life with greater optimism and positivity; (b) Engagement (E), or engaging in interesting activities that stimulate learning new things, which has a positive impact on the development of abilities, intelligence and emotional skills, and increasing happiness; (c) Relationships (R), which are having intense relationships with family members, peers, and colleagues and represent a protective factor for the individual in the most stressful moments of life, allowing the individual to experience greater well-being and feel socially integrated and supported by others; (d) Meaning (M), that is it is important to interpret the events to give a meaning to the existence and everyday experience (e.g., in relation to a religious faith or to the values of a community); and (e) Accomplishments (A), or setting realistic goals and implementing attempts for fulfillment, as realization involves the development of skills and mastery in various sectors such as work, sports, hobbies, etc.

Cultivating these five factors every day can lead to increased mental and physical well-being. In fact, they combine into the concept of Flourishing, which indicates the possibility of experiencing a life characterized by virtue, productivity, growth, and resilience (Keyes et al., 2002), and optimal functioning that includes the realization of human abilities and excellence (Ryan et al., 2010). The interest in prosperity concerns not only the individual and psychological areas, but also the political and organizational, widening the perspectives of this concept (Delle Fave and Massimini, 2005).

The literature shows that variables related to well-being are both contextual, such as need-supportive interpersonal behavior and need-thwarting interpersonal behavior (Yalçın, 2015; Rocchi et al., 2017; Ryan and Deci, 2017), and dispositional, such as positive and negative affectivity (Watson et al., 1988; Kahneman, 1999; Seligman, 2011; Malinowski and Lim, 2015; Sorrenti et al., 2015b; Martino et al., 2019a,b,c), explanatory style (Malhotra and Suri, 2016), dispositional optimism (Liu et al., 2018 (Duy and Yıldız, 2017; Seligman, 1991; Khosroshahi and Abad, 2012; Carver and Scheier, 2014; Ryff, 2014), and academic achievement (Steinmayr et al., 2016; Bücker et al., 2018).

Regarding the contextual factors related to well-being, the literature shows that interpersonal behavior that supports or thwarts choice (autonomy), competence, and relationships could facilitate human flourishing (Chirkov and Ryan, 2001; Vansteenkiste et al., 2010; Vansteenkiste and Ryan, 2013; Rocchi et al., 2017) and constitute protection from the risk of developing illnesses (Yalçın, 2015; Yıldız and Karadaş, 2017). The positive relationship between well-being and supportive interpersonal behavior has been demonstrated (Chirkov and Ryan, 2001; Vansteenkiste et al., 2010; Vansteenkiste and Ryan, 2013; Rocchi et al., 2017), as well as the negative relationship between well-being and thwarted interpersonal behavior (Vansteenkiste et al., 2010; Rocchi et al., 2017; Filippello et al., 2019b). In particular, a supportive context that encourages autonomy increases the level of student engagement and effectively balances students’ needs and their academic activities, promoting the formation of an internal locus of motivation (Reeve et al., 2004; Reeve and Jang, 2006) and persistence even in the face of failure (Aunola et al., 2013). Therefore, a supportive environment strengthens self-realization and can facilitate positive functioning among adolescents within schools (Reeve and Jang, 2006; Filippello et al., 2019a). On the contrary, a thwarting context, characterized by pressure and coercion that makes individuals feel inadequate and emotionally disconnected, favors maladjustment and the development of school HPL (Barber and Harmon, 2002; Soenens et al., 2010; Li et al., 2016; Filippello et al., 2020).

Regarding dispositional variables, while positive affectivity produces enthusiasm, readiness, interest, joy, and determination, negative affectivity triggers suffering related to anxiety, disgust, contempt, hostility and fear, sadness, and HPL (Watson et al., 1988). According to the theory of HPL (Abramson et al., 1989), a repeated condition of uncontrollability to aversive environmental stimuli can foster the conviction of not being able to face the situation. A condition of repeated failures can lead to the belief that attempts to modify events are ineffective, establishing a vicious circle between cognition and behavior (Liu et al., 2015; Sorrenti et al., 2019). HPL can be modulated by three different inferences with respect to negative events: (a) inferences about why the event occurred, linked to causal attributions, in which an internal locus, stable and global, aggravates the desperation; (b) inferences about the consequences of the negative event, that is, consequences that are perceived as more important (for example, the failure of an admissions test to a prestigious university), stable, and with repercussions in many areas of life, the more desperation is generalized and severe; (c) inferences about the self, which are carried out on personal characteristics (e.g., one’s own abilities, personality, social desirability, and the quality of interpersonal relationships – Beck, 1967) that are affected when the perception of immutability of the cause, its stability, and the impact in many areas are greater.

Explanatory style can have a deep impact on an individual’s thinking and affectivity in specific situations, such as when it is necessary to provide an explanation of success or failure (Sorrenti et al., 2015a, 2018). According to the most accredited definitions, an individual may have a locus of internal control (e.g., intelligence, commitment, skill) or an external locus out of his or her control (e.g., situations or environmental conditions) (Weiner et al., 1971). A relevant aspect is the perception of controllability, that is, the belief that a phenomenon may be subject to change and that a greater commitment may or may not change the situation (Weiner, 1985). If an individual is convinced he can modify the events, attributing the cause to unstable internal factors such as commitment, he could be more easily predisposed to dispositional optimism, defined as a global expectation that, in the future, more positive (desirable) than negative (undesirable) events could occur (Scheier and Carver, 1985). An individual with dispositional optimism has an active and proactive attitude toward adversity and implements less avoidance strategies than a pessimistic individual (Segerstrom et al., 2017). It is assumed that dispositional optimism, considered a personality trait, is stable with little possibility of change (Peterson, 2000). Furthermore, it is correlated with the “big five” traits of extroversion and emotional stability (Coelho et al., 2018). Both physical and psychological well-being are positively influenced by dispositional optimism (Carver et al., 2010). In fact, optimistic individuals pay attention to their physical health, implementing healthy behaviors and more functional coping strategies. Furthermore, they are very committed to social relations and, consequently, benefit from this commitment (Carver and Scheier, 2014). On the contrary, subjects with a pessimistic explanatory style, who attribute a negative event to internal, stable, and global causes, have a greater risk of developing negative emotional states that can lead to the development of HPL (Abramson et al., 1989; Liu et al., 2015).

Regarding the relationship between well-being and academic achievement, several studies have shown small positive relations (e.g., Ying and Liese, 1991; Gilman and Huebner, 2006; Murray-Harvey, 2010) while other studies showed no significant correlation (e.g., Liao and Wei, 2014; Bücker et al., 2018), suggesting that students’ academic performance is not necessarily a source of well-being or illness. Furthermore, the direction of the relationship between these two variables is unclear. In fact, while most studies have considered well-being a significant predictor of academic achievement (Fiedler and Beier, 2014; Mega et al., 2014; Bücker et al., 2018), little research considers academic achievement a significant predictor of well-being, indicating that students with high achievements experience greater well-being and low levels of psychopathology (Steinmayr et al., 2016; Neubauer et al., 2017; Ryan and Deci, 2017; Bücker et al., 2018). Moreover, other research has shown that low academic achievement is an important precursor of illness, such as school HPL (Richardson et al., 2005; Huang, 2014; Shek and Li, 2016).

In according with the PERMA model by Seligman (2011), which expands the conceptualization of the construct of “well-being,” and considering the impact of contextual (need-supportive interpersonal behavior and need-thwarting interpersonal behavior) and dispositional variables (affectivity, explanatory style, and optimism) and academic achievement on well-being, there emerges the importance of investigating the role of these factors in promoting well-being and countering the development of psychological problems such as HPL.

## The Present Study

From the studies described above, it is clear that the literature’s interest has been focused on the study of well-being or illness. However, there has been little research that has investigated the relationship between wellness and HPL and the underlying contextual and dispositional variables and academic achievement. Therefore, the aim of the present study was to examine the relationship between contextual variables (need-supportive interpersonal behavior and need-thwarting interpersonal behavior), dispositional variables (dispositional optimism, positive/negative affectivity, explanatory style), academic achievement, general well-being, and school HPL in adolescent students.

Based on the literature, it was hypothesized that general well-being positively correlates with need-supportive interpersonal behavior, dispositional optimism, positive affectivity, adaptive explanatory style, and academic achievement, and that school HPL correlates positively with need-thwarting interpersonal behavior, negative affectivity, and dysfunctional explanatory styles and negatively with academic achievement.

Furthermore, we wanted to investigate the predictive role of contextual and dispositional variables and academic achievement on general well-being and school HPL.

## Materials and Methods

### Participants

The study sample consisted of 218 participants, 116 females (53.2%) and 102 males (46.8%). All participants ranged in age from 16 to 19 years, with a mean of 17.22 years (SD = 0.85). The participants were recruited from a high school in the city of Reggio Calabria within Italy. All participants were Italian citizens and spoke Italian. Regarding their socioeconomic status (SES), 40.5% of the families belonged to the medium SES (one or both parents held a high school diploma) group, 36.7% of the families belonged to the low SES (one or both parents held a lower secondary education diploma) group, and 22.8% belonged to high SES (one or both parents held a university degree) group. Family SES was determined based on the parents’ education, by merging maternal and paternal educational levels into a single category of SES (see Sirin, 2005).

### Instruments

An adapted version of the *Interpersonal Behaviours Questionnaire* (IBQ; Rocchi et al., 2017), was used to evaluate the extent of participants who perceived other people’s interpersonal behaviors as need-supportive or need-thwarting. The questionnaire consisted of 24 items divided into six subscales: autonomy-supportive (e.g., “Support my decisions”), autonomy-thwarting (e.g., “Impose their opinions on me”), competence-supportive (e.g., “Provide valuable feedback”), competence-thwarting (e.g., “Doubt my capacity to improve”), relatedness-supportive (e.g., “Is interested in what I do”), and relatedness-thwarting (e.g., “Do not connect with me”). Participants were asked to indicate to what extent they agreed that “The people in my life…” displayed these behaviors, using a 7-point scale ranging from 1 (Do not agree at all) to 7 (Completely agree). In this study, the total scores of need-supportive and need-thwarting interpersonal behavior were computed by calculating the average of the items that constitute the six subscales and by calculating the average of the items that constitute the autonomy-thwarting, competence-thwarting, and relatedness-thwarting sub-scales, respectively. The IBQ showed good psychometric characteristics (Rocchi et al., 2017). The Italian version of the IBQ was developed using the back-translation method.

The Italian version of the *Life Orientation Test-revised* by Giannini et al. (2008) (LOT-r, Scheier and Carver, 1985) was used to evaluate dispositional optimism. The instrument is composed of 10 items (e.g., “Overall, I expect more good things to happen to me than bad,” “In uncertain times, I usually expect the best,” “I’m always optimistic about my future”). Participants responded on a 5-point Likert-type scale, ranging from 0 (Strongly disagree) to 4 (Strongly agree). The LOT-r demonstrated acceptable reliability and construct validity in previous studies (Giannini et al., 2008; Di Fabio and Bucci, 2015).

The Italian version of the *Positive Affect Scale of the PANAS* by Terraciano et al. (2003) and Watson et al. (1988) was used to evaluate positive and negative affect. The questionnaire consisted of 20 adjectives, of which 10 evaluated the positive affect (e.g., “Interested,” “Enthusiastic,” “Attentive”) and 10 evaluated the negative affect (e.g., “Hostile,” “Afraid,” “Ashamed”). The participants responded on a 5-point Likert-type scale ranging from 1 (Lightly) to 5 (Extremely), referring to the emotions and feelings they had experienced in the last 7 days. The PANAS demonstrated acceptable reliability and construct validity in previous studies (Terraciano et al., 2003; Di Fabio and Bucci, 2015).

An adapted version for the school context of the *Hopelessness Depression Symptom Questionnaire* (HDSQ - Metalsky and Joiner, 1991, unpublished) was used to evaluate HPL in the school context. The instrument is composed of 28 items (e.g., “I can’t do my homework as well as usual”; “I am a burden to teachers or my classmates”; “When I study, my energy is lower than usual”). The participants responded on a 4-point Likert-type scale; the higher the score, the greater the severity of the symptom. The Italian version of the HDSQ was developed using a back-translation method.

The Italian version of the *PERMA-Profiler* (Giangrasso, 2018) was used to assess general well-being. The questionnaire consists of 23 items, 15 of them measuring: positive emotion, engagement, relationships, meaning, and accomplishment along with three independent factors (health, negative emotions, and loneliness), plus a single item that measures happiness. For the present study, 16 items were used (e.g., “How much of the time do you feel you are making progress toward accomplishing your goals?”) that related to the five pillars to measure overall well-being score and the overall happiness item. All items were rated on an 11-point Likert-type scale, with 0 indicating extremely low levels and 10 indicating extremely high levels. Overall well-being score was calculated as the average of 16 items. The PERMA-Profiler demonstrated acceptable reliability and construct validity in previous studies (Butler and Kern, 2016; Giangrasso, 2018).

An adapted version of the *Multidimensional Multiattributional Causality Scale* (MMCS - Lefcourt, 1981) was used to evaluate explanatory style. This self-report questionnaire consists of 48 items divided into two scales (causal attribution in relationships and causal attribution in the school context); both scales can be divided into four types of attributions: internal/stable “attribution to skill” (school context—e.g., “The most important ingredient to getting good grades is my academic ability”; relationships—e.g., “It seems to me that getting along with people is a skill”), internal/unstable “attribution to commitment” (school context—e.g., “In my case, the good grades I receive are always the direct result of my efforts”; relationships—e.g., “Maintaining friendships requires real effort to make them work”), external/stable “attribution to context” (school context—e.g., “Often my poorer grades are obtained in courses that the professor has failed to make interesting”; relationships—e.g., “No matter what I do, some people just don’t like me”), and external/unstable “attribution to luck” (school context—e.g., “Sometimes my success on the exams depends on some luck”; relationships—e.g., “Often chance events can play a large part in causing rifts between friends”). In this study, only two subscales relating to attribution to commitment in the school context and attribution to luck in the school context were used. The participants responded on a 5-point Likert-type scale ranging from 0 (Strongly disagree) to 4 (Strongly agree). The MMCS demonstrated acceptable reliability and construct validity in previous studies (Lefcourt et al., 1984; Ngunu et al., 2019). The Italian version of the MMCS was developed using a back-translation method.

### Procedure

The parents of the underaged students were asked to provide informed consent for their participation in the study. The older students participated in the study voluntarily after signing the informed consent form. The students completed the questionnaire in their classrooms during school hours in a single session. The instructions stated that the questionnaires were voluntary and the identity of the respondents and their individual responses would be kept confidential. Completion of the questionnaire took 20–30 min. The protocol was approved by the Ethics Committee of the Centre for Research and Psychological Intervention (CERIP) of the University of Messina (protocol number: 30465).

### Data Analysis

The Statistical Package for the Social Sciences (SPSS 19) was used to conduct descriptive statistics, Cronbach’s alpha, correlations, and hierarchical regression.

## Results

### Descriptive Statistics and Correlations

Table 1 shows the means, standard deviations, skewness, kurtosis, Cronbach’s alpha values, and correlations for all measures considered in this study. A descriptive analysis showed that all scales had good scores for symmetry and kurtosis, and the reliability of the measures was adequate.

**TABLE 1:** Descriptive Statistics and Correlation among measures.

	M	SD	Skew	Kurt	1	2	3	4	5	6	7	8	9	10
(1) Need supportive Interpersonal Behavior	5.43	0.98	–0.72	0.51	α = 0.89									
(2) Need twarthing Interpersonal Behavior	3.76	0.17	0.93	0.14	−0.54**	α = 0.88								
(3) Dispositional optimism	2.09	0.70	–0.28	–0.37	0.18**	−0.20**	α = 0.67							
(4) Positive affectivity	3.31	0.62	–0.33	0.00	0.32**	–0.07	0.48**	α = 0.76						
(5) Negative affectivity	2.41	0.75	0.41	–0.39	−0.23**	0.36**	−0.54**	−0.27**	α = 0.81					
(6) Attribution commitment school	2.84	0.61	–0.20	–0.40	0.14*	–0.04	0.23**	0.26**	−0.21**	α = 0.63				
(7) Attribution luck school	1.69	0.71	–0.20	0.08	–0.05	0.15*	–0.11	–0.13	0.13	−0.19**	α = 0.69			
(8) Academic achievement	7.30	0.91	0.16	0.06	0.10	–0.02	0.05	0.09	0.01	0.10	−0.17*	−		
(9) General well-being	7.01	1.4	–0.73	0.33	0.53**	−0.36**	0.51**	0.50**	−0.46**	0.31**	–0.07	0.15*	α = 0.86	
(10) School hopelessness	1.94	0.42	0.88	0.73	−0.21**	0.34**	−0.41**	−0.31**	0.53	−0.30**	0.28**	−0.28**	−0.44**	α = 0.89

*N* = 218; **p < 0.01; *p < 0.05.

The correlations showed that general well-being was positively related with need-supportive interpersonal behavior, dispositional optimism, positive affectivity, attribution to commitment in the school context, and academic achievement, while it was negatively correlated with need-twarthing interpersonal behavior, negative affectivity, and school HPL. Meanwhile, school HPL was positively related with need-twarthing interpersonal behavior, negative affectivity, and attribution to luck in the school context, though it was negatively correlated with need-supportive interpersonal behavior, dispositional optimism, positive affectivity, attribution to commitment in the school context, and academic achievement.

### Regression Analyses

#### Regression Analyses on Well-Being

Gender, age and SES were entered in Step 1 as control variables. Need-supportive and need-thwarting interpersonal behavior were entered in Step 2. Dispositional optimism, positive affectivity, negative affectivity, attribution to commitment in the school context, and attribution to luck in the school context were entered in Step 3 and academic achievement in Step 4.

The first step was not significant, *F*(3,214) = 2.12, *p* > 0.05, explained 3% of the variance in well-being. In the second step, there was a significant change in *R*^2^ [*F* change (2,212) = 45.16; *p* < 0.001, *R*^2^ change = 0.29] and the model explained an additional 29% of the variance in well-being, *F* (5,212) = 19.86; *p* < 0.001, *R*^2^ adj = 0.30, with gender significantly negative predictor being of general well-being, while need-supportive interpersonal behavior was significantly positive predictor of general well-being. In the third step, there was a significant change in *R*^2^ [*F* change (5,207) = 18.89; *p* < 0.001, *R*^2^ change = 0.21] and the model explained an additional 21% of the variance in well-being, *F*(10,207) = 23.56; *p* < 0.001, *R*^2^ adj = 0.51, with need-supportive interpersonal behavior remaining a significant positive predictor of general well-being, followed by dispositional optimism, positive affectivity and attribution to commitment in the school context, while negative affectivity was a significant negative predictor of general well-being. In the fourth step, where academic achievement was entered into the regression, there was not a significant change in *R*^2^ [*F* change (1, 206) = 3.28; *p* > 0.05, *R*^2^ change = 0.01], explained 1% of the variance in well-being, *F* (11, 206) = 21.95; *p* < 0.001, *R*^2^ adj = 0.52, with need-supportive interpersonal behavior, dispositional optimism, positive affectivity, attribution to commitment in school context and negative affectivity maintaining unique contributions (Table 2).

**TABLE 2:** Hierarchical regression analysis for general well-being and school hoplessness.

	General Well-being		School Hopelessness	
	**t**	**β**	**t**	**β**

**(1) Step**	***R*^2^ = *0.03***		***R*^2^ = *0.02***	

Gender	–1.20	–0.08	0.79	0.05
Age	0.92	0.06	0.22	0.01
SES	1.73	0.12	–1.63	–0.11

**(2) Step**	*R*^2^ = *0.32***Δ***R^2^* = 0.29***		*R^2^* = *0.13***Δ***R^2^* = 0.11***	

Gender	–2.24	−0.13*	0.94	0.06
Age	0.65	0.04	0.25	0.02
SES	1.43	0.08	–1.16	–0.08
Need-Supportive Interpersonal Behavior	7.31	0.50***	–0.62	–0.05
Need-Thwarthing Interpersonal Behavior	–1.12	–0.08	3.98	0.31***

**(3) Step**	*R*^2^ = *0.53***Δ***R*^2^ = 0.21***		*R*^2^ = *0.40***Δ***R^2^* = 0.27***	

Gender	–0.08	0.00	–0.29	–0.02
Age	–0.11	–0.01	1.30	0.07
SES	0.78	0.04	–0.71	–0.04
Need-Supportive Interpersonal Behavior	5.61	0.35***	0.74	0.05
Need-Thwarthing Interpersonal Behavior	–0.78	–0.05	2.64	0.18**
Dispositional optimism	3.37	0.21***	–1.07	–0.08
Positive affectivity	3.44	0.20***	–1.91	–0.13
Negative affectivity	–2.82	−0.17**	5.14	0.36***
Attribution commitment school	2.57	0.13**	–2.54	−0.15**
Attribution luck school	0.92	0.05	2.60	0.15**

**(4) Step**	***R*^2^ = *0.54***Δ***R*^2^ = *0.01***		*R*^2^ = *0.45***Δ***R*^2^ = 0.05***	

Gender	–0.23	–0.01	0.05	0.00
Age	0.22	0.01	0.56	0.03
SES	0.86	0.04	–0.91	–0.05
Need-Supportive Interpersonal Behavior	5.48	0.34***	1.11	0.07
Need-Thwarthing Interpersonal Behavior	–0.86	–0.05	2.91	0.20***
Dispositional optimism	3.32	0.21***	–0.95	–0.07
Positive affectivity	3.38	0.20***	–1.82	–0.12
Negative affectivity	–2.88	−0.18***	5.47	0.37***
Attribution commitment school	2.48	0.13**	–2.39	−0.13*
Attribution luck school	1.14	0.06	2.15	0.12*
Academic achievement	1.81	0.09	–4.25	−0.23***

*N* = 218; ***p < 0.001; **p < 0.01; *p < 0.05.

#### Regression Analyses on School Hopelessness

Gender, age and SES were entered in Step 1 as control variables. Need-supportive and need-thwarting interpersonal behavior were entered in Step 2. Dispositional optimism, positive affectivity, negative affectivity, attribution to commitment in the school context, and attribution to luck in the school context were entered in Step 3 and academic achievement in Step 4.

The first step was not significant, *F*(3,214) = 1.32, *p* > 0.05, explained 2% of the variance in well-being. In the second step, there was a significant change in *R*^2^ [*F* change (2, 212) = 13.40; *p* < 0.001, *R*^2^ change = 0.11] and the model explained an additional 11% of the variance in school HPL, *F* (5,212) = 6.24; *p* < 0.001, with need-thwarting interpersonal behavior being a significant positive predictor of school HPL. In the third step, there was a significant change in *R*^2^ (*F* change (5,207) = 18.95; *p* < 0.001, *R*^2^ change = 0.27) and the model explained an additional 27% of the variance in school HPL, *F* (10,207) = 13.92; *p* < 0.001, *R*^2^ adj = 0.37, with need-thwarting interpersonal behavior remaining a significant positive predictor of school HPL, followed by negative affectivity and attribution of luck in the school context, while attribution to commitment in the school context was a significant negative predictor of school HPL. Finally, in the fourth step, there was a significant change in *R*^2^ [*F* change (1, 206) = 18.06; *p* < 0.001, *R*^2^ change = 0.05], explained 5% of the variance in school HPL, *F* (11,206) = 15.34; *p* < 0.001, *R*^2^ adj = 0.42, with need-thwarting interpersonal behavior, negative affectivity, attribution of luck in the school context, and attribution to commitment in school context maintaining unique contribution and academic achievement providing an additional unique contribution (Table 2).

## Discussion

The study’s results showed the existence of a relation between contextual and dispositional variables, academic achievement, general well-being, and school HPL in adolescent students. As expected and consistent with PERMA theory (Seligman, 2011), the interpersonal context play an important role in the promotion of general well-being and the reduction of discomfort through the satisfaction of psychological basic needs of autonomy, competence and relatedness. In particular, our results suggest that the students’ perceptions of need-supportive interpersonal behaviors, that encouraging the choice, self-regulation, and perspective of the other (Sheldon and Filak, 2008; Mageau et al., 2015; Rocchi et al., 2017) underlines competence, and that expressed interest, facilitates human flourishing (Chirkov and Ryan, 2001; Vansteenkiste et al., 2010; Vansteenkiste and Ryan, 2013; Rocchi et al., 2017). Considering that the developmental of optimal functioning (flourishing) is constituted by the possibility of the students engaging in interesting activities and consistent with own values, to experience meaningful relationships and to implementing attempts for fulfilment that involves the development of skills and mastery in various sectors (Seligman, 2011), perception of another person’s behavior in a need-supportive of autonomy, competence and relatedness way could promote general well-being. In fact, need-supportive interpersonal behavior refers to a wider environmental feature able to create a generally supportive climate, than it refers to the perception of the subject to receive support in the most stressful moments (Rocchi et al., 2017). Furthermore, our data showed that students who expect positive outcomes to occur, experience positive affects, and attribute their success to commitment experience a greater degree of well-being than students who experience negative affects, consistent with previous studies (Carver et al., 2010; Di Fabio and Bucci, 2015; Segerstrom et al., 2017). In addition, unlike what was hypothesized, academic achievement did not predicted general well-being, suggesting that although academic achievement is significant because it shapes a person’s life chances it does not have such a strong influence on student’s well-being (Liao and Wei, 2014; Bücker et al., 2018). Instead, general well-being is more influenced by dispositional variables and in particular by the perception of an interpersonal context, supporting the needs of autonomy, competence, and relationships, which is consistent with previous studies (Chirkov and Ryan, 2001; Vansteenkiste et al., 2010; Vansteenkiste and Ryan, 2013; Liao and Wei, 2014; Steinmayr et al., 2016; Rocchi et al., 2017; Bücker et al., 2018).

On the contrary, our results suggest that an interpersonal context, thwarting the needs of autonomy, competence, and relationships, through interpersonal behaviors characterized by pressure and coercion that makes students feel inadequate and emotionally disconnected (Vansteenkiste et al., 2010; Rocchi et al., 2017), favors the development of school HPL (Barber and Harmon, 2002; Soenens et al., 2010; Li et al., 2016). More specifically, students who perceive a hindering environmental climate could do negative inferences about the own personal characteristics, such as one’s own abilities, personality, and the quality of interpersonal relationships (Beck, 1967), that could be affecting hopelessness feeling. Furthermore, our results suggest that feelings of school HPL are positively affected by experimenting unhealthy emotions and attributing the causes of school-related events to uncontrollable rather than controllable events, fostering a belief in not being able to face the situation and the belief that attempts to modify events are ineffective, according to Abramson et al. (1989) and Liu et al. (2015). Finally, academic achievement was a significant and negative predictor of school HPL. Students who experience repeated failures in school could increase the perception of the inability to reach their important goals, rising school HPL. This result is consistent with previous studies that indicate that academic achievement is an important precursor of HPL (Richardson et al., 2005; Huang, 2014; Shek and Li, 2016).

Overall, the findings of this study indicate that similar but opposite factors influence students’ perception of well-being and feelings of school HPL. Although the results confirm the reference literature about the predictive role of dispositional variables, an interesting result is related to the predictive role of students’ perception of interpersonal behavior on students’ well-being and illness. In fact, to our knowledge, this is the first study that considers the interpersonal context in terms of perception of another people’s behavior in a need-supportive or need-thwarting of autonomy, competence, and relatedness manner. This suggests that a supportive school climate can foster the realization of students’ potential and optimal functioning (flourishing), while a hindering school climate can lead to the development of school HPL.

The present study does suffer from some limitations. First, the study was cross-sectional in nature; therefore, further studies could use a longitudinal design to further examine dynamic changes in the relationships between analyzed variables. Moreover, the sample of this study was represented by students from a single Italian region; future research should extend these results to more heterogeneous samples to explore the generalizability of the findings.

Despite these limitations, this study could have positive practical implications. It would be useful to structure targeted training to favor psychological well-being. For example, students should be helped to develop appropriate personal beliefs and good strategies of self-regulation to prevent the risk of developing emotionally distressful conditions. In addition, it is essential that teachers use supportive interpersonal behaviors and transmit them to their students to increase functional models in the school setting and in life in general. This would make the school environment a place that promotes well-being and a better quality of life.

International literature shows many examples of this type of training arising from positive psychology and aimed at promoting well-being in the school context, such as, for example, the “Strath Haven Positive Psychology Curriculum” (see Peterson and Seligman, 2004; Dahlsgaard et al., 2005), the “Penn Resiliency Program” (see Seligman et al., 2009; Vahedi et al., 2016), and the “Geelong Grammar School for Positive Education” (see Norrish et al., 2013).”

From studies on positive psychology, a new key of interpretation is born, that of not acting in a preventive perspective but with a view to promote well-being. Considering the aspects of general well-being as opposed to those characterizing HPL, there is the possibility of decreasing the risk of illness and allowing each individual to “flourish” (Keyes et al., 2002). For decades, positive psychology has been committed to promoting the potential and optimal functioning (flourishing) of all individuals – not just students – by structuring special training. Stimulate (1) positive emotions, (2) engagement in interesting activities, (3) intense relationships with others, (4) the ability to give meaning to the existence and everyday experience, and (5) the ability to set realistic settings goals and implementing attempts for fulfillment is increasingly important today. In this historical moment, the challenge facing psychology is to guarantee people’s psychological wellbeing and to promote resilience and optimal functioning that includes the realization of human abilities and excellence (Ryan et al., 2010).

## Data Availability Statement

The datasets generated for this study are available on request to the corresponding author.

## Ethics Statement

The studies involving human participants were reviewed and approved by the Ethics Committee of the Centre for Research and Psychological Intervention (CERIP) of the University of Messina (protocol number: 30465). Written informed consent to participate in this study was provided by the participants’ legal guardian/next of kin.

## Author Contributions

CB assisted with the study concept, manuscript preparation, and data analysis. LS assisted with the manuscript preparation, editing, and study design. SO assisted with the manuscript preparation, and data collection. DM assisted with the study design and data collection. PF assisted with the manuscript preparation, study design, interpretation of results, and study supervision. All authors contributed to the article and approved the submitted version.

## Conflict of Interest

The authors declare that the research was conducted in the absence of any commercial or financial relationships that could be construed as a potential conflict of interest.

## References

[B1] Abramson L. Y. Metalsky G. I. Alloy L. B. (1989). Hopelessness depression: a theory-based subtype of depression. *Psychol. Rev.* 96 358–372. 10.1037/0033-295X.96.2.358

[B2] Aunola K. Viljaranta J. Lehtinen E. Nurmi J. E. (2013). The role of maternal support of competence, autonomy and relatedness in children’s interests and mastery orientation. *Learn. Individ. Differ.* 25 171–177. 10.1016/j.lindif.2013.02.002

[B3] Barber B. K. Harmon E. L. (2002). “Violating the self: parental psychological control of children and adolescents,” in *Intrusive Parenting: How Psychological Control Affects Children and Adolescents*, ed. Barber B. K. (Washington, DC: American Psychological Association), 15–52. 10.1037/10422-002

[B4] Beck A. T. (1967). *Depression: Clinical, Experimental and Theoretical Aspects.* New York, NY: Harper and Row.

[B5] Bücker S. Nuraydin S. Simonsmeier B. A. Schneider M. Luhmann M. (2018). Subjective well-being and academic achievement: a meta-analysis. *J. Res. Pers.* 74 83–94. 10.1016/j.jrp.2018.02.007

[B6] Butler J. Kern M. L. (2016). The PERMA-Profiler: a brief multidimensional measure of flourishing. *Int. J. Well Being* 6 1–48. 10.5502/ijw.v6i3.526 32285354

[B7] Carver C. S. Scheier M. F. (2014). Dispositional optimism. *Trends Cogn. Sci.* 18 293–299. 10.1016/j.tics.2014.02.003 24630971 PMC4061570

[B8] Carver C. S. Scheier M. F. Segerstrom S. C. (2010). Optimism. *Clin. Psychol. Rev.* 30 879–889. 10.1016/j.cpr.2010.01.006 20170998 PMC4161121

[B9] Chirkov V. I. Ryan R. M. (2001). Parent and teacher autonomy-support in Russian and US adolescents: common effects on well-being and academic motivation. *J. Cross Cult. Psychol.* 32 618–635. 10.1177/0022022101032005006

[B10] Coelho G. L. H. Vilar R. Hanel P. H. P. Monteiro R. P. Ribeiro M. G. C. Gouveia V. V. (2018). Optimism scale: evidence of psychometric validity in two countries and correlations with personality. *Pers. Individ. Dif.* 134 245–251. 10.1016/j.paid.2018.06.030

[B11] Dahlsgaard K. Peterson C. Seligman M. E. P. (2005). Shared virtue: the convergence of valued human strengths across culture and history. *Rev. Gen. Psychol.* 9 203–213. 10.1037/1089-2680.9.3.203

[B12] Delle Fave A. Massimini F. (2005). The investigation of optimal experience and apathy: developmental and psychosocial implications. *Eur. Psychol.* 10 264–274. 10.1027/1016-9040.10.4.264

[B13] Di Fabio A. Bucci O. (2015). Affective profiles in Italian high school students: life satisfaction, psychological well-being, self-esteem, and optimism. *Front. Psychol.* 6:1310. 10.3389/fpsyg.2015.01310 26388814 PMC4554945

[B14] Duy B. Yıldız M. (2017). The mediating role of self-esteem in the relationship between optimism and subjective well-being. *Curr. Psychol.* 38 1456–1463. 10.1007/s12144-017-9698-1

[B15] Fiedler K. Beier S. (2014). “Affect and cognitive processes in educational contexts,” in *International Handbook of Emotions in Education*, eds Pekrun R. Linnenbrink-Garcia L. Pekrun R. Linnenbrink-Garcia L. (New York, NY: Routledge), 36–55.

[B16] Filippello P. Buzzai C. Costa S. Orecchio S. Sorrenti L. (2020). Teaching style and academic achievement: the mediating role of learned helplessness and mastery orientation. *Psychol. Sch.* 57 5–16. 10.1002/pits.22315 33136418

[B17] Filippello P. Buzzai C. Costa S. Sorrenti L. (2019a). School refusal and absenteeism: perception of teacher behaviors, psychological basic needs, and academic achievement. *Front. Psychol.* 10:1471. 10.3389/fpsyg.2019.01471 31316431 PMC6610479

[B18] Filippello P. Buzzai C. Messina G. Mafodda A. V. Sorrenti L. (2019b). School refusal in students with low academic performances and Specific Learning Disorder. The role of self-esteem and perceived parental psychological control. *Intl. J. Disabil. Dev. Educ.* 1–30. 10.1080/1034912X.2019.1626006

[B19] Giangrasso B. (2018). Psychometric properties of the PERMA-Profiler as hedonic and eudaimonic well-being measure in an Italian context. *Curr. Psychol.* 1–10. 10.1007/s12144-018-0040-3

[B20] Giannini M. Schuldberg D. Di Fabio A. Gargaro D. (2008). Misurare l’ottimismo: proprietà psicometriche della versione Italiana del Life Orientation Test—Revised (LOT-R) [Measuring optimism: psychometric properties of the Italian version of the Life Orientation Test—Revised (LOT-R)]. *Counseling* 1 73–84.

[B21] Gilman R. Huebner E. S. (2006). Characteristics of adolescents who report very high life satisfaction. *J. Youth Adolesc.* 35 311–319.

[B22] Huang H. (2014). *Social Media Generation in Urban China: A Study of Social Media use and Addiction Among Adolescents.* Berlin: Springer-Verlag.

[B23] Kahneman D. (1999). “Objective happiness,” in *Well-Being: the Foundations of Hedonic Psychology*, eds Kahneman D. Diener E. Schwarz N. (New York, NY: Russell Sage), 3–25.

[B24] Keyes C. L. M. Shmotkin D. Ryff C. D. (2002). Optimizing well-being: the empirical encounter of two traditions. *J. Pers. Soc. Psychol.* 82 1007–1022. 10.1037/0022-3514.82.6.1007 12051575

[B25] Khosroshahi J. B. Abad T. H. N. (2012). The relationship between social anxiety, optimism and self-efficacy with psychological well-being in students. *J. Urmia Univ. Med. Sci.* 23 115–122.

[B26] Lefcourt H. M. (1981). The construction and development of the multidimensional-multiattributional causality scales. *Res. Locus Control Construct* 1 245–277. 10.1016/b978-0-12-443201-7.50011-7

[B27] Lefcourt H. M. Martin R. A. Ware E. E. (1984). Locus of control, causal attributions, and affects in achievement-related contexts. *Can. J. Behav. Sci. Rev. Can. Sci. Comport.* 16 57–64. 10.1037/h0080771

[B28] Li D. Li X. Wang Y. Bao Z. (2016). Parenting and Chinese adolescent suicidal ideation and suicide attempts: the mediating role of hopelessness. *J. Child Fam. Stud.* 25 1397–1407. 10.1007/s10826-015-0334-0

[B29] Liao K. Y. Wei M. (2014). Academic stress and positive affect: Asian value and self worth contingency as moderators among Chinese international students. *Cultur. Divers. Ethnic Minor. Psychol.* 20 107–115. 10.1037/a0034071 24491130

[B30] Liu C. Cheng Y. Hsu A. S. C. Chen C. Liu J. Yu G. (2018). Optimism and self-efficacy mediate the association between shyness and subjective well-being among Chinese working adults. *PLoS One* 13:e0194559. 10.1371/journal.pone.0194559 29668678 PMC5905885

[B31] Liu R. T. Kleiman E. M. Nestor B. A. Cheek S. M. (2015). The hopelessness theory of depression: a quarter century in review. *Clin. Psychol.* 22 345–365. 10.1111/p.12125 PMC4689589 26709338

[B32] Mageau G. A. Ranger F. Joussemet M. Koestner R. Moreau E. Forest J. (2015). Validation of the perceived parental autonomy support scale (P-PASS). *Can. J. Behav. Sci. Rev. Can. Sci. Comport.* 47 251–262. 10.1037/a0039325

[B33] Malhotra R. Suri S. (2016). Locus of control & well-being among college students. *Indian J. Behav. Sci.* 1 1–19.

[B34] Malinowski P. Lim H. J. (2015). Mindfulness at work: positive affect, hope, and optimism mediate the relationship between dispositional mindfulness, work engagement, and well-being. *Mindfulness* 6 1250–1262. 10.1007/s12671-015-0388-5

[B35] Martino G. Bellone F. Langher V. Caputo A. Catalano A. Quattropani M. C. (2019a). Alexithymia and psychological distress affect perceived quality of life in patients with type 2 diabetes mellitus. *Mediterr. J. Clin. Psychol.* 7 1–15. 10.6092/2282-1619/2019.7.2328 31160582

[B36] Martino G. Catalano A. Bellone F. Russo G. T. Vicario C. M. Lasco A. (2019b). As time goes by: anxiety negatively affects the perceived quality of life in patients with type 2 diabetes of long duration. *Front. Psychol.* 10:1779. 10.3389/fpsyg.2019.01779 31428028 PMC6689992

[B37] Martino G. Langher V. Cazzato V. Vicario C. M. (2019c). Psychological factors as determinants of medical conditions. *Front. Psychol.* 10:2502. 10.3389/fpsyg.2019.02502 31781002 PMC6857525

[B38] Mega C. Ronconi L. De Beni R. (2014). What makes a good student? How emotions, self-regulated learning, and motivation contribute to academic achievement. *J. Educ. Psychol.* 106 121–131. 10.1037/a0033546

[B39] Murray-Harvey R. (2010). Relationship influences on students’ academic achievement, psychological health and well-being at school. *Educ. Child Psychol.* 27 104–115.

[B40] Neubauer A. B. Lerche V. Voss A. (2017). Inter-individual differences in the intra-individual association of competence and well-being: combining experimental and intensive longitudinal designs. *J. Pers.* 86 698–713. 10.1111/jopy.12351 28888049

[B41] Ngunu S. Kinai T. Ndambuki P. Mwaura P. (2019). Causal attributions as correlates of secondary school students’ academic achievement. *Educ. Res. Int.* 2019:1950753 10.1155/2019/1950753

[B42] Norrish J. M. Williams P. O’Connor M. Robinson J. (2013). An applied framework for positive education. *Int. J. Wellbeing* 3 147–161. 10.5502/ijw.v3i2.2 32285354

[B43] Peterson C. (2000). The future of optimism. *Am. Psychol.* 55 44–55. 11392864 10.1037//0003-066x.55.1.44

[B44] Peterson C. Seligman M. E. P. (2004). *Character Strengths and Virtues: A Handbook and Classification.* Washington, DC: American Psychological Association.

[B45] Reeve J. Jang H. (2006). What teachers say and do to support students’ autonomy during a learning activity. *J. Educ. Psychol.* 98 209–218. 10.1037/0022-0663.98.1.209

[B46] Reeve J. Jang H. Carrell D. Jeon S. Barch J. (2004). Enhancing students’ engagement by increasing teachers’ autonomy support. *Motiv. Emot.* 28 147–169. 10.1023/B:MOEM.0000032312.95499.6f

[B47] Richardson A. S. Bergen H. A. Martin G. Roeger L. Allison S. (2005). Perceived academic performance as an indicator of risk of attempted suicide in young adolescents. *Arch. Suicide Res.* 9 163–176. 10.1080/13811110590904016 16020160

[B48] Rocchi M. Pelletier L. Cheung S. Baxter D. Beaudry S. (2017). Assessing need-supportive and need-thwarting interpersonal behaviours: the Interpersonal Behaviours Questionnaire (IBQ). *Pers. Individ. Dif.* 104 423–433. 10.1016/j.paid.2016.08.034

[B49] Ryan R. M. Deci E. L. (2017). *Self-Determination Theory: Basic Psychological Needs in Motivation, Development, and Wellness.* New York, NY: Guilford Publications.

[B50] Ryan R. M. Weinstein N. Bernstein J. Brown K. W. Mistretta L. Gagne M. (2010). Vitalizing effects of being outdoors and in nature. *J. Environ. Psychol.* 30 159–168. 10.1016/j.jenvp.2009.10.009

[B51] Ryff C. D. (2014). Psychological well-being revisited: advances in the science and practice of eudaimonia. *Psychother. Psychosom.* 83 10–28. 10.1159/000353263 24281296 PMC4241300

[B52] Scheier M. F. Carver C. S. (1985). Optimism, coping, and health: assessment and implications of generalized outcome expectancies. *Health Psychol.* 4 219–247. 10.1037/0278-6133.4.3.219 4029106

[B53] Segerstrom S. C. Carver C. S. Scheier M. F. (2017). “Optimism,” in *The Happy Mind: Cognitive Contributions to Well-Being*, eds Robinson M. Eid M. (Cham: Springer), 195–212.

[B54] Seligman M. (1991). *Learned Optimism: How to Change Your Mind and Your Life.* New York, NY: Pocket Books.

[B55] Seligman M. (2011). *Flourish.* New York, NY: Simon & Schuster.

[B56] Seligman M. E. Ernst R. M. Gillham J. Reivich K. Linkins M. (2009). Positive psychology and classroom interventions. *Oxf. Rev. Educ.* 35 293–311. 10.1080/03054980902934563

[B57] Shek D. T. Li X. (2016). Perceived school performance, life satisfaction, and hopelessness: a 4-year longitudinal study of adolescents in Hong Kong. *Soc. Indic. Res.* 126 921–934. 10.1007/s11205-015-0904-y 26912946 PMC4751166

[B58] Sheldon K. M. Filak V. (2008). Manipulating autonomy, competence, and relatedness support in a game-learning context: new evidence that all three needs matter. *Br. J. Soc. Psychol.* 47 267–283. 10.1348/014466607X238797 17761025

[B59] Sirin S. R. (2005). Socioeconomic status and academic achievement: a meta-analytic review of research. *Rev. Educ. Res.* 75 417–453. 10.3102/00346543075003417

[B60] Soenens B. Vansteenkiste M. Luyten P. (2010). Toward a domain-specific approach to the study of parental psychological control: distinguishing between dependency-oriented and achievement-oriented psychological control. *J. Pers.* 78 217–256. 10.1111/j.1467-6494.2009.00614.x 20433618

[B61] Sorrenti L. Filippello P. Buzzai C. Buttò C. Costa S. (2018). Learned helplessness and mastery orientation: the contribution of personality traits and academic beliefs. *Nord. Psychol.* 70 71–84. 10.1080/19012276.2017.1339625

[B62] Sorrenti L. Filippello P. Buzzai C. Costa S. (2015a). A psychometric examination of the Learned Helplessness Questionnaire in a sample of Italian school students. *Psychol. Sch.* 52 923–941. 10.1002/pits.21867

[B63] Sorrenti L. Filippello P. Buzzai C. Costa S. (2015b). Tolleranza alla frustrazione e benessere psicologico: quale relazione? [Frustration tolerance and psychological well-being: what relationship?]. *Psicol. Salute* 3 65–86. 10.3280/PDS2015-003004

[B64] Sorrenti L. Spadaro L. Mafodda A. V. Scopelliti G. Orecchio S. Filippello P. (2019). The predicting role of school Learned helplessness in internalizing and externalizing problems. An exploratory study in students with Specific Learning Disorder. *Mediterr. J. Clin. Psychol.* 7 1–14.

[B65] Steinmayr R. Crede J. McElvany N. Wirthwein L. (2016). Subjective well-being, test anxiety, academic achievement: testing for reciprocal effects. *Front. Psychol.* 6:1994. 10.3389/fpsyg.2015.01994 26779096 PMC4705295

[B66] Terraciano A. McCrae R. R. Costa P. T. Jr. (2003). Factorial and construct validity of the Italian Positive and Negative Affect Schedule (PANAS). *Eur. J. Psychol. Assess.* 19 131–141. 10.1027//1015-5759.19.2.131 20467578 PMC2868265

[B67] Vahedi S. Gargari R. B. Gholami S. (2016). Effect of the penn resiliency program on student with emotional problems. *Int. J. Educ. Psychol. Res.* 2 145–149. 10.4103/2395-2296.180302

[B68] Vansteenkiste M. Niemiec C. P. Soenens B. (2010). “The development of the five mini-theories of self-determination theory: an historical overview, emerging trends, and future directions,” in *The Decade Ahead: Theoretical Perspectives on Motivation and Achievement*, eds Urdan T. Karabenick S. (Bingley: Emerald Group Publishing Limited), 105–165. 10.1108/s0749-7423(2010)000016a007

[B69] Vansteenkiste M. Ryan R. M. (2013). On psychological growth and vulnerability: basic psychological need satisfaction and need frustration as a unifying principle. *J. Psychother. Integr.* 23 263–280. 10.1037/a0032359

[B70] Watson D. Clark L. A. Tellegen A. (1988). Development and validation of brief measures of positive and negative affect: the PANAS scales. *J. Pers. Soc. Psychol.* 54 1063–1070. 10.1037/0022-3514.54.6.1063 3397865

[B71] Weiner B. (1985). An attributional theory of achievement motivation and emotion. *Psychol. Rev.* 92 548–573. 10.1037/0033-295x.92.4.548 3903815

[B72] Weiner B. Frieze I. H. Kukla A. Reed L. Rest S. Rosenbaum R. M. (1971). *Perceiving the Causes of Success and Failure.* Morristown, NJ: General Learning Press.

[B73] Yalçın Ý. (2015). Ýyi oluş ve sosyal destek arasındaki ilişkiler: türkiye’de yapılmış çalışmaların meta analizi [Relationships between well-being and social support: a meta-analysis of studies conducted in Turkey]. *Türk. Psikiyatr. Derg.* 26 21–32. 25742034

[B74] Yıldız M. Karadaş C. (2017). Multiple mediation of self-esteem and perceived social support in the relationship between loneliness and life satisfaction. *J. Educ. Pract.* 8 130–139.

[B75] Ying Y. W. Liese L. H. (1991). Emotional well-being of Taiwan students in the US: an examination of pre-to post-arrival differential. *Int. J. Intercult. Relat.* 15 345–366. 10.1016/0147-1767(91)90007-4

